# Effects of promyelocytic leukemia zinc finger protein on the proliferation of cultured human corneal endothelial cells

**Published:** 2007-04-27

**Authors:** Takeshi Joko, Daisuke Nanba, Fumio Shiba, Kazunori Miyata, Atsushi Shiraishi, Yuichi Ohashi, Shigeki Higashiyama

**Affiliations:** 1 Department of Ophthalmology, Ehime University Graduate School of Medicine, Shitsukawa, Toon, Ehime, Japan; 2 Department of Biochemistry and Molecular Genetics, Ehime University Graduate School of Medicine, Shitsukawa, Toon, Ehime, Japan; 3 Miyata Eye Hospital, Miyakonojo, Miyazaki, Japan; 4 Precursory Research for Embryonic Science and Technology, Japan Science and Technology Agency, 4-1-8 Honcho Kawaguchi, Saitama, Japan

## Abstract

**Purpose:**

To determine whether the promyelocytic leukemia zinc finger (PLZF) protein, a transcriptional repressor and negative regulator during cell cycling, plays a role in the proliferation of cultured human corneal endothelial cells (HCECs).

**Methods:**

The expressions of the mRNA and the protein of PLZF were determined by real-time PCR and western blot analysis, respectively. The changes in the expression of the PLZF gene of cultured HCECs were investigated at different times after cell-cell contacts were disrupted by incubation with EDTA. The cell proliferation rate was assessed with a real-time cell electronic sensing (RT-CES) system after cultured HCECs were infected with either PLZF or LacZ encoding adenovirus vector (Ad-PLZF or Ad-LacZ). The PLZF-regulating genes were analyzed by DNA microarray analysis in cultured HCECs infected with Ad-PLZF.

**Results:**

The expression of the mRNA of PLZF was first detected when the cultured HCECs became confluent, and the relative amount of PLZF mRNA continued to increase for up to 5 days as the cell-cell contacts were formed more firmly. On the other hand, the expression of the mRNA of PLZF decreased about 20 fold 3 h after EDTA exposure, and gradually returned to the original level as the cell-cell contacts were reformed at 72 h after the exposure. The assessment using the RT-CES system showed that the proliferation of cultured HCECs was inhibited for up to 72 h when infected by Ad-PLZF. DNA microarray analysis revealed that the transforming growth factor-β stimulated clone 22 *(TSC-22)* gene was up-regulated by 2.32 fold when infected by Ad-PLZF.

**Conclusions:**

These findings indicate that the expression of PLZF in HCECs is closely related to the formation of cell-cell contacts, and that PLZF may play a role in suppressing their proliferation.

## Introduction

The corneal endothelium is a single layer of cells lining the posterior surface of the cornea, and it helps maintain corneal transparency by regulating the hydration of the corneal stroma. It is widely accepted that corneal endothelial cells do not proliferate in humans once the endothelial monolayer is formed [1]. It is also known that the density of human corneal endothelial cells (HCECs) decreases by approximately 0.5% per year throughout life, and that the enlargement of the remaining cells compensates for this reduction to maintain corneal endothelial function [1-4]. In addition, a variety of injuries such as surgical stress during intraocular surgery, corneal trauma, and viral infections often cause an extensive reduction in the number of corneal endothelial cells, which can result in corneal endothelial dysfunction known as bullous keratopathy. Although penetrating keratoplasty (PKP) and other modern surgical procedures, including deep lamellar endothelial keratoplasty (DLEK), stripping and automated endothelial keratoplasty (DSAEK), can restore vision in such patients, it would be more beneficial to regulate the proliferation of corneal endothelial cells by manipulating cell cycles.

It has been demonstrated that HCECs are arrested in the G_1_ phase of the cell cycle in vivo rather than resting in the G_0_ phase [5]. In fact, HCECs have been shown to possess a strong potential to proliferate depending upon the age they are cultured [6]. Among the anti-mitotic factors associated with the G_1_ arrest of the HCECs, transforming growth factor-β2 (TGF-β_2_) is known to block the G_1_-S transition by blocking the phosphorylation of p27^kip1^, which is required for the nuclear export of the inhibitory molecules for degradation [7,8]. The mechanisms involved in the cell cycling and proliferation of HCECs however have not been fully determined as yet.

In searching for a cell cycling mechanism, we focused on DNA binding transcriptional factors, including the members of the BTB/POZ-zinc finger protein family. The BTB/POZ-zinc finger proteins are sequence-specific transcriptional repressors characterized by a BTB/POZ domain, which is responsible for transcriptional repression, and a zinc finger domain that forms the DNA binding domain [9]. Among the family members, the *PLZF* gene was first identified by its fusion to the retinoid acid receptor (RAR) alpha locus in a therapy-resistant form of acute promyelocytic leukemia associated with the t(11;17) translocation [10]. *PLZF* is a sequence-specific DNA binding transcriptional repressor that suppresses the transcription of genes such as *cyclin A2* and *c-myc* [11-13]. In addition to these genes, it has been reported that pre-B-cell leukemia transcription factor 1 (Pbx1) can be a target gene for PLZF to suppress melanoma cell growth [14].

In preliminary experiments, we investigated the expression pattern of several members of the BTB/POZ-zinc finger protein family that were considered to be negative regulators in the cycling of HCECs. Interestingly, the expression level of the mRNA of PLZF alone was found to vary according to the state of the cell-cell contact. Thus, the purpose of this study is to investigate the sequential changes in the expression of the mRNA of PLZF in HCECs in the primary culture and after EDTA exposure. In addition, we examined what role PLZF plays in the proliferation of HCECs using an adenovirus vector carrying genes encoding PLZF.

## Methods

### Media and culture conditions

All primary and passaged HCECs were cultured in a media consisting of Dulbecco modified Eagle medium (DMEM) supplemented with 15% fetal bovine serum (FBS), 30 mg/l of L-glutamine, 2.5 mg/l of Fungizone (GIBCO, Grand Island, NY), 2.5 mg/l of doxycycline (Sigma-Aldrich Co., St.Louis, MO), and 2 μg/ml of basic fibroblast growth factor (Invitrogen, Carlsbad, CA) [15]. Cultured HCECs were maintained in a humidified incubator at 37 °C and 10% CO_2_.

### Primary cultures of human corneal endothelial cells

All procedures including those on human subjects were conducted in accordance with the principles of the Declaration of Helsinki [16], and this study was approved by the Institutional Review Board of Ehime University.

Primary cultures of HCECs were started from normal human corneas acquired from the American Eye Bank. Human tissue was used in strict accordance with the tenets of the Declaration of Helsinki. The corneoscleral buttons were stored in Optisol (Chiron, Irvine, CA) at 4 °C and were cultured within 10 days of enucleation. Small explants from the endothelial layer, including Descemet's membrane, were removed with sterile surgical forceps and cultured in 35 mm culture dishes coated with mouse collagen type IV (BD Biosciences, San Jose, CA).

When a sufficient density of proliferating cells was attained, the cultured HCECs were rinsed three times in Ca^2+^- and Mg^2+^-free phosphate-buffered saline (PBS^-^), trypsinized for 2 min at 37 °C, and passaged at ratios ranging from 1:1 to 1:4, depending on the number of proliferating colonies [15]. All subsequent passages were carried out using the same method, but at a ratio of 1:6. The approximate time to confluence after each passaging was 6 to 8 days. We used cultured human corneal endothelial cells at the fifth passage for the experiments.

### RNA extraction and RT-PCR

Total RNA was isolated from HCECs and cultured HCECs using TRIzol reagent according to the manufacturer's instructions (Invitrogen). They were further purified by RNeasy kit (Qiagen, Valencia, CA). cDNA was synthesized with Superscript II reverse transcriptase according to the manufacturer's instructions (Invitrogen).

PCR amplification was performed with TaKaRa Ex Taq (TaKaRa, Kusatsu, Japan) under the following conditions: 94 °C for 5 min, 35 or 40 cycles of denaturation at 94 °C for 10 s, annealing at 60 °C, except for Kaiso (62 °C for Kaiso), for 20 s, and extension at 72 °C for 30 s. The primer pairs used for RT-PCR are listed in Table 1.

**Table 1:** Sequences of primers used in RT-PCR.

**Primer**	**Sequence (5'-3')**	**Product size**	**GenBank number**
GAPDH	F1-CGTATTGGGCGCCTGGTCACCAG	294 bp	AY340484
GAPDH	R1-TCACTCCTGGAAGATGGTGATGGG		
PLZF	F1-CCACCCCTACGAGTGTGAGT	181 bp	NM_006006
PLZF	R1-CTCAAAGGGCTTCTCACCTG		
BCL-6	F1-GATGAGATTGCCCTGCATTT	203 bp	NM_138931
BCL-6	R1-TTCTTCCAGTTGCAGGCTTT		
Kaiso	F1-ACCTGTGCAGGAATTTCCAC	221 bp	AY302699
Kaiso	R1-GAGCGGCCAAGTTACTGAAG		
MYNN	F1-AGGCCAAGCCAATGTGTAAC	248 bp	AY514901
MYNN	R1-ATGATGCATGCGACTATGGA		
KIAA0441	F1-CTTGTTGGGGATCAAGAGGA	214 bp	NM_014797
KIAA0441	R1-GGACCTGTAGCGAGTGCTTC		
ZNF278	F1-AAGCAGGTGGCTTGTGAGAT	174 bp	NM_032052
ZNF278	R1-CCACAGCTCTGGCAGATGTA		
E-cadherin	F1-TGCCCAGAAAATGAAAAGG	200 bp	NM_004360
E-cadherin	R1-GTGTATGTGGCAATGCGTTC		
N-cadherin	F1-GACAATGCCCCTCAAGTGTT	179 bp	NM_001792
N-cadherin	R1-CCATTAAGCCGAGTGATGGT		
VE-cadherin	F1-CCTACCAGCCCAAAGTGTGT	249 bp	NM_001795
VE-cadherin	R1-GACTTGGCATCCCATTGTCT		
V-cadherin	F1-TGATGATGCCAAAAACCTCA	198 bp	NM_001257
V-cadherin	R1-ATGGGCAGGTTGTAGTTTGC		
P-cadherin	F1-AACCTCCACAGCCACCATAG	181 bp	NM_001793

In the table, GAPDH=glyceral-dehyde-3-phosphate dehydrogenase, PLZF=promyelocytic leukemia zinc finger, and BCL-6 =B-cell lymphoma 6.

### Real-time polymerase chain reaction

Real-time PCR was performed with the DyNAmo STBR Green qPCR kit (Finnzymes, Espoo, Finland) under the following conditions: 95 °C for 15 min, 40 cycles of denaturation at 95 °C for 10 s, annealing at 60 °C for 20 s, and extension at 72 °C for 30 s using the Opticon2 DNA Engine (Bio Rad, Hercules, CA). The primer pairs used for real time PCR are listed in Table 2. The C_t_ values were determined by Opticon2 software, and the amount of each mRNA was calculated relative to the amount of GAPDH mRNA in the same samples [17]. Each run was completed with a melting curve analysis to confirm the specificity of the amplification and the absence of primer dimers.

**Table 2:** Sequences of primers used in real-time PCR.

**Primer**	**Sequence (5'-3')**	**Product size**	**GenBank number**
GAPDH	F2-CGACCACTTTGTCAAGCTCA	228 bp	AY340484
GAPDH	R2-AGGGGTCTACATGGCAACTG		
PLZF	F2-GGTCGAGCTTCCTGATAACG	237 bp	NM_006006
PLZF	R2-GCCATGTCAGTGCCAGTATG		
TSC-22	F1-GCTGCCGTTTTCTGTTTCTC	152 bp	AF256226
TSC-22	R1-ATCCATCGCCACTGGTCTAC		
N-cadherin	F1-GACAATGCCCCTCAAGTGTT	179 bp	NM_001792
N-cadherin	R1-CCATTAAGCCGAGTGATGGT		
ZO-1	F1-TGAGGCAGCTCACATAATGC	224 bp	NM_003257
ZO-1	R1-GGTCTCTGCTGGCTTGTTTC		

In the table, TSC-22=transforming growth factor-β stimulated clone 22.

### Releasing model of cell-cell contacts

To examine the effect of cell-cell contact on the expression of the *PLZF* gene, we first determined the effective concentration of EDTA that altered the integrity of endothelial cell-cell contact. Cultured HCECs were incubated for 2 h in 2.7, 3.2, and 4.0 mM of di-sodium EDTA.2H_2_O (EDTA). EDTA was prepared in DMEM with 15% FBS. Exposure to 2.7 mM of EDTA for 2 h caused a mild lateral separation of the cells, and incubation in 3.2 mM EDTA for 2 h caused a moderate lateral separation of the cells. Incubation in 4.0 mM of EDTA for 2 h caused a marked lateral separation and loss of contact to the dish. When 3.2 mM EDTA was used for 2 h to disrupt the cell-cell contacts of HCECs, the lateral separation of the cells was reversed by replacing the EDTA-rich media with normal culture medium. Therefore, we decided to use 3.2 mM EDTA for 2 h as the condition to disrupt the cell-cell contacts.

HCECs were cultured until confluent, and then the medium was replaced by one containing 3.2 mM EDTA. Cultured HCECs were treated with EDTA for 2 h and returned to the normal culture medium for up to 72 h. Cultured HCECs were collected at 1, 3, 6, 24, and 72 h after returning the cells to the normal culture medium, and the relative amounts of the mRNA of PLZF were evaluated by real-time PCR.

### Adenovirus vector construction and infection into cultured human corneal endothelial cells

The degree of infection by GFP-expressing adenovirus vector (Ad-GFP) of the cultured HCECs was determined by counting the number of GFP positive cells when infected at a multiplicity of infection (MOI) of 50, 100, and 200. Adenovirus vector carrying genes encoding PLZF (Ad-PLZF) or LacZ (Ad-LacZ) were prepared using an adenovirus expression vector kit (Takara Biomedicals) as described [14,18]. Purified, concentrated, and titer-checked viruses were used for the infections.

### Western blot analysis

HCECs in culture dishes were rinsed two times with PBS^-^ and then lysed with Laemmli sample buffer (Bio-Rad, Hercules, CA) with β-mercaptoethanol, and the final concentration was 5%. Equivalent volumes of samples were separated on 7.5% polyacrylamide gel containing sodium dodecyl sulfate (SDS-PAGE) and transferred to polyvinylidene (PVDF) membranes. After blocking with 5% nonfat dry milk and 0.1% Tween-20 in PBS, the membrane was incubated with monoclonal anti-human PLZF antibody (Oncogene Research Products, Cambridge, MA) for 1 h. The positive immunoreactions were made visible by the ECL plus detection system according to the manufacturer's instructions (Amersham Pharmacia Biotech, Piscataway, NJ).

### In vitro cell proliferation

The rate of cellular proliferation was analyzed with a real-time cell electronic sensing (RT-CES) system (ACEA Bioscience, San Diego, CA). Cells were grown on the surfaces of microelectronic sensors, which are composed of a circle-on-line electrode arrays and are integrated into the bottom surfaces of the microtiter plate. Changes in cell number were monitored and quantified by detecting sensor electrical impedance. For cell quantification and viability measurements, the data generated on the RT-CES system correlated well with those from the colorimetric (MTT) assay. Cell index (CI) values obtained on the RT-CES system were quantitatively correlated with the cell numbers [19,20].

The cells were harvested 24 h after infection with Ad-PLZF or Ad-LacZ at an MOI of 100 and seeded into a 16-well strip at a density of 1x10^4^ cells/well. The sensor devices were placed into the 5% CO_2_ incubator, and the cell index value was determined every hour automatically by the RT-CES system for up to 72 h.

### Microarray analyses

Cultured HCECs of 50% confluency were infected with Ad-PLZF or Ad-LacZ at an MOI of 100. Total RNA was isolated from Ad-PLZF or Ad-LacZ infected HCECs at 48 h post-infection. The Acegene Human oligo chip 30K (Hitachi Software Engineering, Yokohama, Japan) containing 30,000 genes was used to compare gene expression in cultured HCECs infected with Ad-PLZF or Ad-LacZ. Initial data analysis for each chip was performed using DNASIS-Array software (Hitachi Software Engineering, Japan).

### Statistical analyses

Values are presented as means±standard deviations. Differences between the groups were analyzed with unpaired Student's t tests. All t tests were two-side, and a p value of <0.05 was considered to be statistically significant.

## Results

### Comparison of mRNA expression of BTB/POZ-zinc finger-containing transcription factors in confluent and subconfluent cultured HCECs in vitro and normal HCECs in vivo

**Figure 1:**
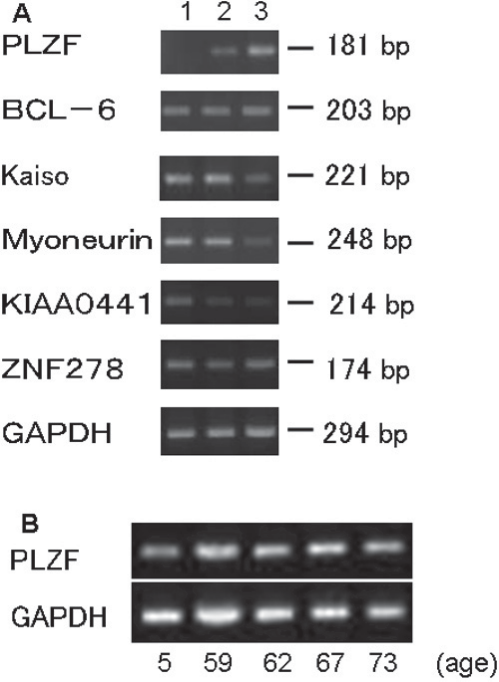
Expression of the mRNA of BTB/POZ-zinc finger-containing transcription factors by RT-PCR. **A**: Ethidium-bromide-stained agarose gels showing PCR products for PLZF, BCL-6, Kaiso, myoneurin, KIAA0441, ZNF278, and GAPDH. Similar results were obtained in two other experiments (35 cycles). Lane 1: Sub-confluent cultured HCECs; Lane 2: 100% confluent cultured HCECs; Lane 3: in vivo normal HCECs. **B**: Ethidium-bromide-stained agarose gels showing PCR products for PLZF in vivo normal human corneas, ages: 5, 59, 62, 67, and 73 years (40 cycles).

The expression of six human BTB/POZ-Zinc finger-containing transcription factors: promyelocytic leukemia zinc finger (PLZF), B-cell lymphoma 6 (BCL-6), Kaiso, myoneurin, KIAA0441, and zinc finger protein278 (ZNF278), were compared in confluent and subconfluent cultured HCECs by RT-PCR. The gene with a different expression pattern might be considered to be a good candidate for regulating the proliferation of HCECs. The results showed that the mRNA of PLZF was expressed only when HCECs were confluent and the expression of the mRNA of the other transcription factors was unchanged in both confluent and subconfluent HCECs. The mRNA of PLZF was also undetectable by RT-PCR up to 40 cycles in subconfluent cultured HCECs (Figure 1A). These results were consistent with the findings in all HCECs from different human donors. The mRNA of PLZF was also found to be expressed in the corneal endothelial cells obtained from normal human corneas (ages: 5, 59, 62, 67, and 73 years; Figure 1B).

### Kinetics of PLZF mRNA expression in primary cultured HCEC

**Figure 2:**
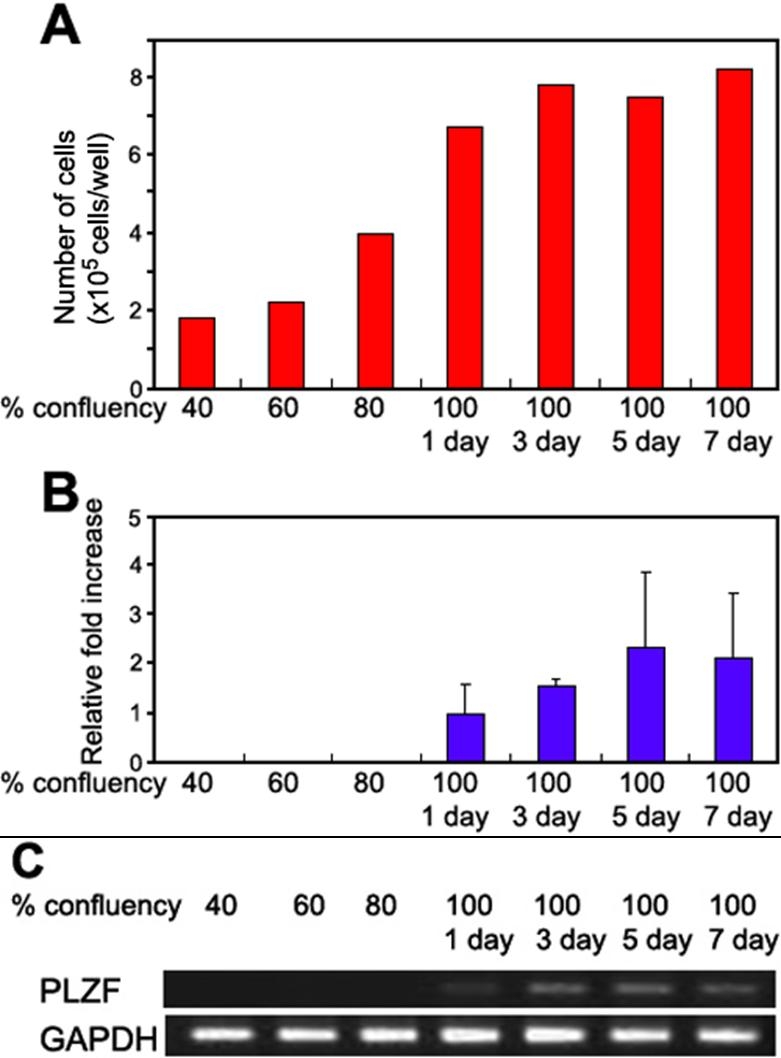
Kinetics of mRNA of PLZF in primary cultured HCECs. **A**: Cell number in 6 well plates for each condition. **B**: Time course of the expression of PLZF mRNA in cultured HCECs. Cultured HCECs were collected at 40%, 60%, 80%, and 100% (1 day, 3 days, 5 days, and 7 days after reached confluency). The relative expression of PLZF mRNA was determined by real-time PCR, and the amount of each mRNA was calculated relative to the amount of GAPDH mRNA in the same sample (n=3 each). The ratio of the sample from 100%/1 day was set to "1". **C**: Ethidium-bromide-stained agarose gels showing PCR (40 cycles) products for PLZF and GAPDH.

Next, the kinetics of the PLZF mRNA expression in primary cultured HCECs was examined by real-time PCR. The mRNA of PLZF was not expressed when HCECs were still in the proliferation phase but began to be expressed at the 100% confluency (1 day) which seemed to occur when they reached confluency (Figure 2B). The mRNA of PLZF was undetectable by RT-PCR up to 40 cycles in cultured HCECs of 40%, 60%, and 80% confluency (Figure 2C). After reaching confluency, the relative expression level of PLZF mRNA continued to increase for up to 5 days (Figure 2A-C). Cell-cell contacts were still not formed in the proliferation phase, but were formed after the 100% confluency. These results led us to hypothesize that the PLZF expression might be related to cell-cell contact and should be investigated in more detail.

### Effect of disruption of the cell-cell contact on PLZF mRNA expression

**Figure 3:**
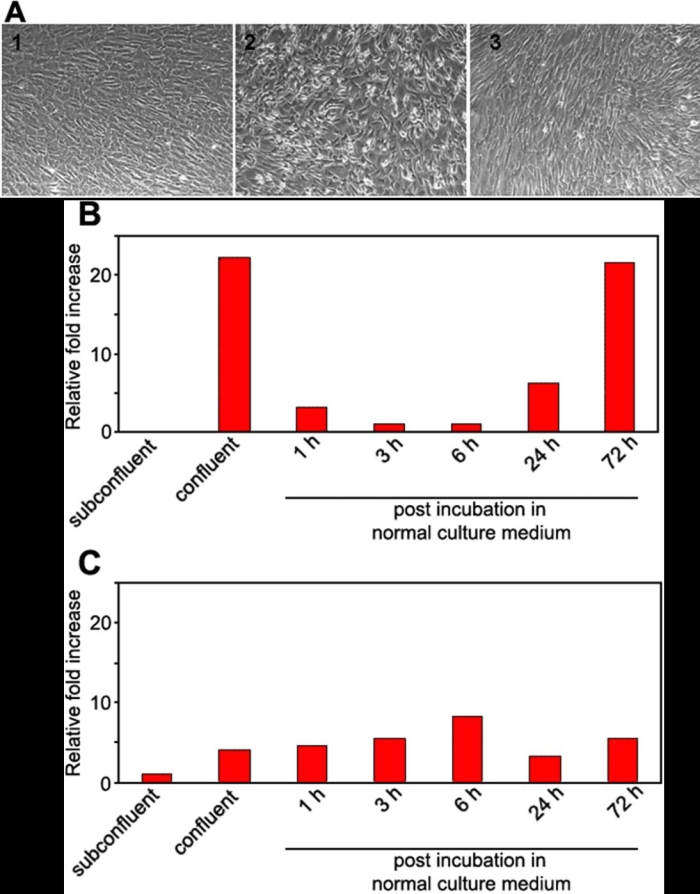
Effect of disruption of the cell-cell contact on PLZF mRNA expression. **A**: Effect of 3.2 mM of EDTA on the integrity of human corneal endothelial cell-cell contact. 1: No treatment: cell-cell contact is intact. 2: Incubation with EDTA for 2 h leads to moderate cell-cell separation. 3: Incubation in medium without EDTA for 24 h after incubation with EDTA for 2 h. Cell-cell contact has recovered. **B**: HCECs were cultured until confluent, the medium was then replaced by medium containing 3.2 mM EDTA for 2 h, and returned to the normal culture medium for 72 h. The relative expression of PLZF mRNA was determined by real-time PCR, and the amount of mRNA was calculated relative to the amount of GAPDH mRNA in the same sample. The ratio of the sample from 3 h post-incubation was set to 1. This experiment was repeated twice. **C**: The same experiment was performed on cultured human umbilical vein endothelial cells. The ratio of the sample from subconfluent culture was set to 1.

To determine whether cell-cell contact is associated with the expression of the *PLZF* gene, confluently-cultured HCECs were incubated with 3.2 mM EDTA for 2 h and replaced with normal culture medium containing no EDTA (Figure 3A). After the treatment, HCECs were harvested at specific times, and the changes of the mRNA of PLZF were assessed by real-time PCR. The results showed that the expression of the mRNA of PLZF decreased by about 20 fold at 3 h after the EDTA treatment, but began to increase at 24 h after replacing the EDTA media with normal media. The level of PLZF recovered to the original level when the cell-cell contacts were reformed at 72 h (Figure 3B).

When the same experiment was performed on cultured human umbilical vein endothelial cells (HUVEC), the expression of PLZF mRNA was not changed by EDTA treatment (Figure 3C), suggesting the importance of PLZF in the cell-cell contact of corneal endothelial cells.

### Effect of PLZF gene transfer on the proliferation of cultured HCECs

**Figure 4:**
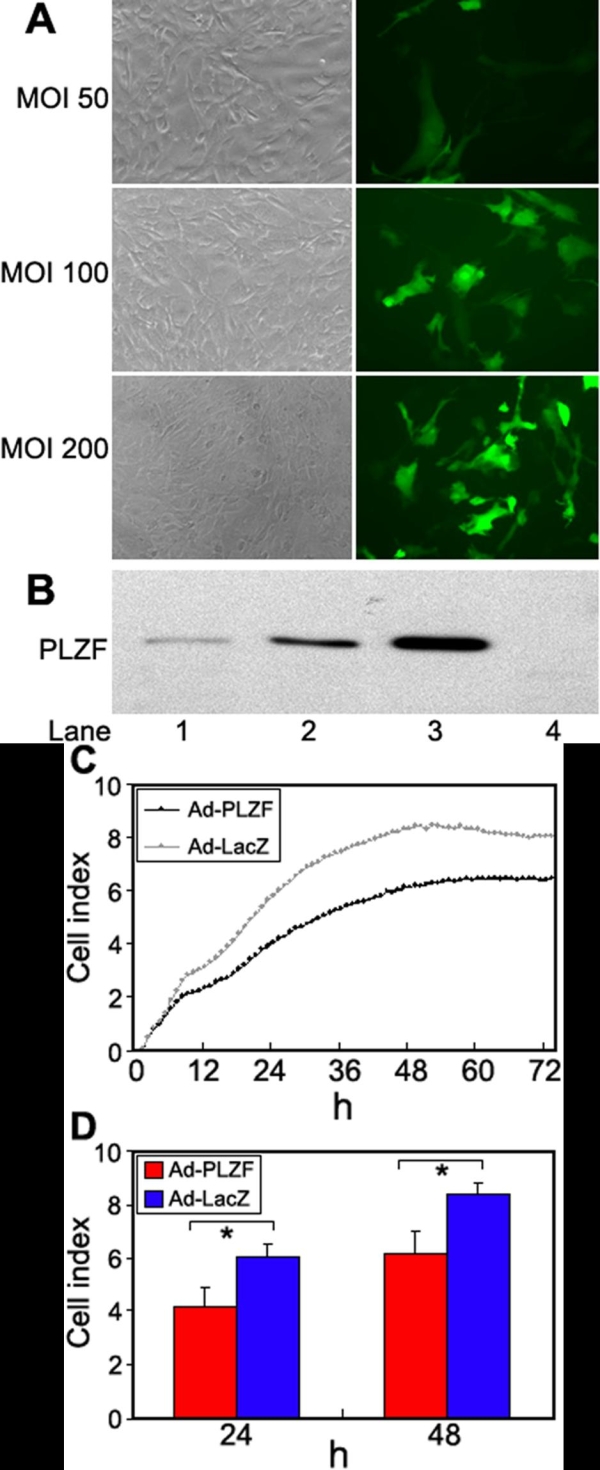
Efficiency of adenovirus vector infection and effect of PLZF on proliferation of cultured HCECs. **A**: GFP-positive cells in cultured HCECs 48 h after infection. HCECs at 80% confluency were infected at an MOI of 50, 100, and 200 with an adenovirus vector expressing GFP. Phase-contrast photograph (left) and fluorescence photograph (right) showing the infection efficiency was 14% at an MOI of 50, 24% at an MOI of 100, and 41% at an MOI of 200. **B**: Western blot analysis with anti-PLZF antibody of cultured HCECs infected with Ad-PLZF and uninfected cells. HCECs at 80% confluency were infected with Ad-PLZF. The cells were harvested 48 h after infection. Lane 1: MOI 50; Lane 2: MOI 100; Lane 3: MOI 200; Lane 4: uninfected HCECs. **C**: The cells were harvested at 24 h after infection, and the cells were seeded into a 16-well strip. Cell index values were determined every hour automatically by the RT-CES system for up to 72 h. Each blot is an average of 8 samples. **D**: Cell index values at 24 and 48 h are shown. Error bars designate standard deviations. The asterisk indicates a significant difference (p<0.001) between Ad-PLZF and Ad-LacZ (n=8 each).

To test the hypothesis that the PLZF may suppress the proliferation of HCECs, we first infected subconfluent cultured HCECs with Ad-PLZF at an MOI of 50, 100, and 200. The efficiency of infection of the Ad-GFP vectors into HCECs at 48 h was 14% at an MOI of 50, 24% at an MOI of 100, and 41% at an MOI of 200 (Figure 4A). At the same time, western blot analysis using anti-PLZF antibody revealed a single band of approximately 70 kDa. The intensity of the bands was strongly correlated to the multiplicity of infection (Figure 4B). Because PLZF mRNA was expressed at low levels in the uninfected cultured HCECs, PLZF protein may be undetectable by the western blot analysis employed in this study.

The cell proliferation assay was done using the RT-CES system with the Ad-LacZ cells serving as the control. When the cell proliferation rate was continuously monitored, it was found that infection with Ad-PLZF at an MOI of 100 inhibited the cell growth by 30.3% at 24 h and 26.5% at 48 h compared to that with Ad-LacZ (Figure 4D). This inhibition was statistically significant, and the effect lasted up to 72 h (Figure 4C).

### Comparison of mRNA expression of cadherin family in cultured HCECs in vitro and normal HCECs in vivo, and kinetics of N-cadherin mRNA expression in primary cultured HCEC

**Figure 5:**
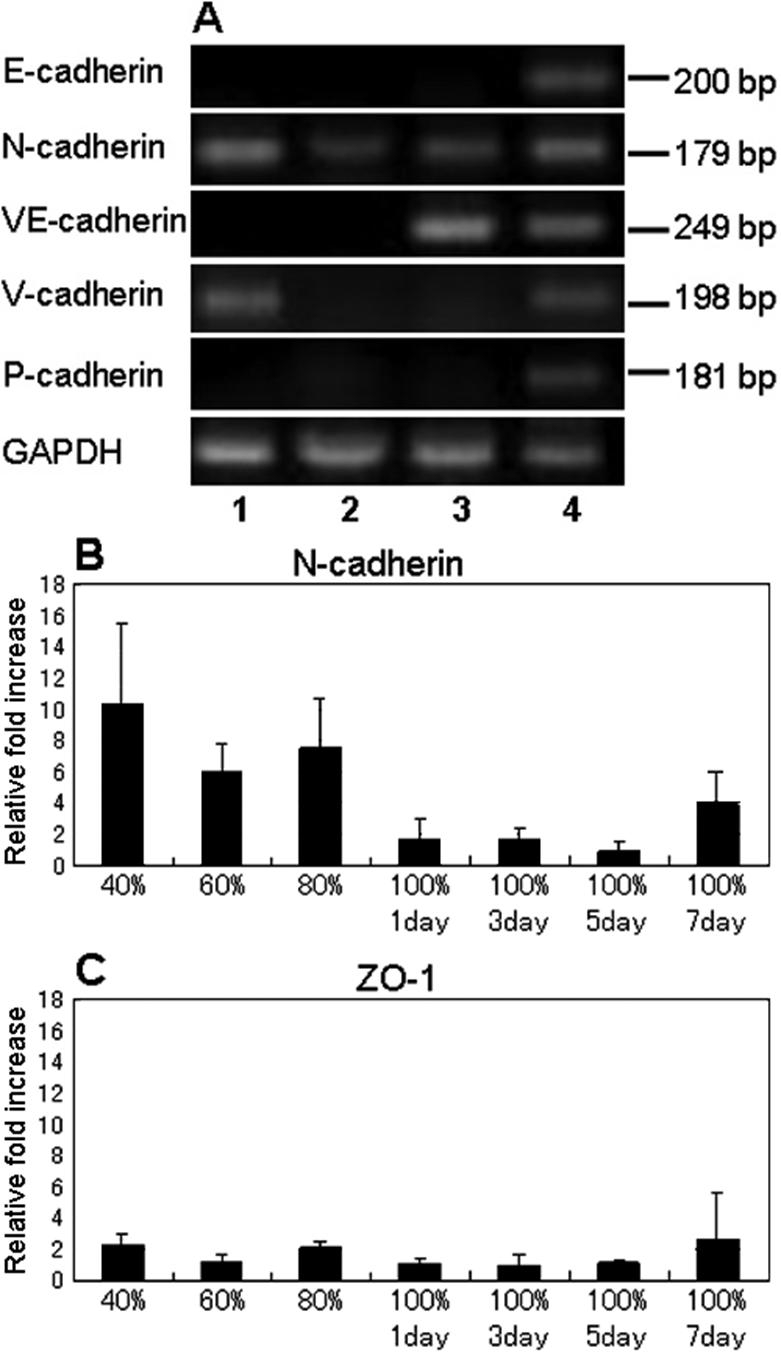
The expression of mRNA of cadherin family and ZO-1. **A**: Ethidium-bromide-stained agarose gels showing PCR products for cadherin family (40 cycles). 1: In vitro HCECs, 2: in vivo HCECs, 3: in vitro HUVEC, 4: normal human skin. **B**, and **C**: Time course of the expression of the N-cadherin and ZO-1 mRNA in cultured HCECs. Cultured HCECs were collected at 40%, 60%, 80%, and 100% (1 day, 3 days, 5 days, and 7 days, respectively) after reaching confluency. The relative expressions of N-cadherin and ZO-1 mRNA were determined by real-time PCR, and the amount of each mRNA was calculated relative to the amount of GAPDH mRNA in the same sample (n=3 each). The ratio of the sample from 100%/5 day was set to 1 for N-cadherin and 100%/3 day to 1 for ZO-1.

The expression of five members of the human cadherin family: E-cadherin, N-cadherin, VE-cadherin, V-cadherin, and P-cadherin, were compared in vitro HCECs, in vivo HCECs, in vitro HUVEC, and normal human skin by RT-PCR. N-cadhein and V-cadherin were detected in in vitro HCECs, whereas only N-cadherin was detected in in vivo HCECs (Figure 5A).

Next, the kinetics of the expression of the mRNA of the N-cadherin was examined in primary cultured HCECs by real-time PCR. In contrast to the expression of PLZF, N-cadherin expression was detected at high levels in subconfluent HCECs, and decreased as cultured HCECs attained confluency (Figure 5B). When the same experiment was performed on the mRNA expression of ZO-1, no change was found in the kinetics of ZO-1 mRNA expression (Figure 5C).

### Determination of PLZF-induced gene expression in HCECs

**Figure 6:**
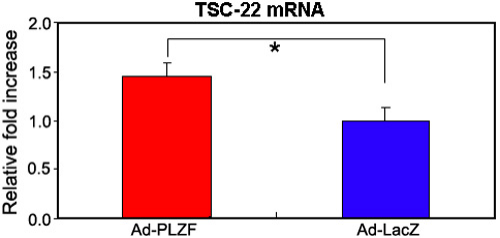
Expression of TSC-22 mRNA in cultured HCECs. HCECs at 50% confluency were infected with Ad-PLZF or Ad-LacZ at an MOI of 100. The cells were harvested 48 h after infection. The relative expression of TSC-22 mRNA was determined by real-time PCR, and the amount of each mRNA was calculated relative to the amount of GAPDH mRNA in the same samples. The ratio of the sample from Ad-LacZ was set to "1". The asterisk indicates a significant difference (p<0.005) between Ad-PLZF and Ad-LacZ (n=4 each).

Finally, the changes in the expression of genes in HCECs induced by PLZF were determined by DNA microarray analysis. When a total of 30,000 genes were analyzed, PLZF was shown to upregulate at least 54 genes and down-regulate at least 34 genes. Unexpectedly, the expression of *cyclin A2* and *c-myc* genes were not affected by an over-expression of the PLZF gene in HCECs (data not shown). Two growth factors including heparin-binding epidermal growth factor-like growth factor (HB-EGF) and connective tissue growth factor were the most down-regulated by 0.059 fold and 0.188 fold, respectively. Of particular interest was the discovery that the transforming growth factor β stimulated clone 22 *(TSC-22)* gene was up-regulated by 2.32 fold (data not shown). The increased expression of the mRNA of TSC-22 was also confirmed by real-time PCR (Figure 6).

### Expression of PLZF mRNA in corneal endothelial cells with iridocorneal endothelial syndrome (ICE syndrome)

**Figure 7:**
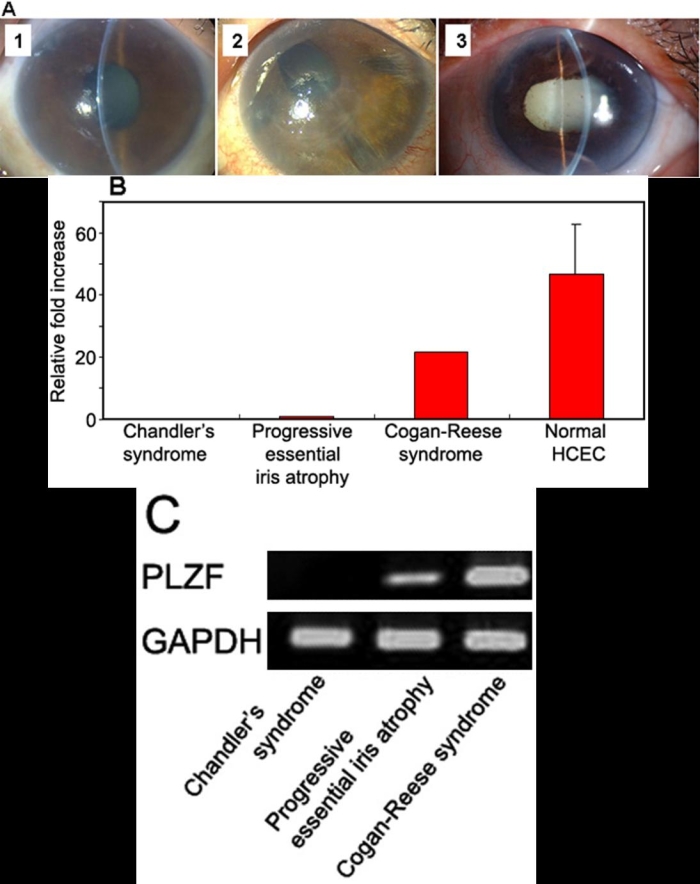
Expression of the mRNA of PLZF in the corneal endothelial cells from patients with the ICE syndrome. **A**: 1: Chandler's syndrome, 2: Progressive essential iris atrophy, 3: Cogan-Reese syndrome. **B**: The relative expression of PLZF mRNA was determined by real-time PCR, and the amount of each mRNA was calculated relative to the amount of GAPDH mRNA in the same samples. The ratio of the sample from progressive essential iris atrophy was set to "1". **C**. Ethidium-bromide-stained agarose gels showing PCR products for PLZF (40 cycles).

We have examined the expression of the mRNA of PLZF in corneal endothelial cells obtained from three patients with the ICE syndrome (Figure 7A), and found that the relative expression of the mRNA of PLZF was lower in these patients than in normal controls by real-time PCR (Figure 7B). In particular, the expression of PLZF mRNA was undetectable in Chandler's syndrome (Figure 7B,C). The relative expression of the mRNA of PLZF as normal controls is the average value of four normal human corneal endothelial cells (ages 62, 67, 73, and 73 years).

## Discussion

PLZF is a transcriptional repressor and is known to suppress the expression of several genes that regulate cell proliferation. Thus, in earlier studies, an enhanced expression of PLZF led to the suppression of proliferation in some cell lines [11,21]. In murine 32Dcl3 cells, the cell cycling profiles of over-expressing PLZF were significantly altered, and up to 80% of the cells accumulated in the G_0_/G_1_ phase with a significantly smaller proportion of cells than in the S phase [21].

Our results showed that, among the different members of the BTB/POZ-zinc finger family, PLZF was the only gene that varied in association with the alterations of HCECs. Thus, PLZF was not expressed in HCECs in the proliferative phase and was later expressed when the cultured cells reached confluency. We also found that the expression of the PLZF mRNA was profoundly decreased when the cell-cell contact was disrupted by EDTA treatment, and mRNA expression returned to the original level as the cell-cell contact was reformed. These changes in the expression pattern suggest that PLZF gene expression may be regulated by cell-cell contact and related to the proliferation of HCECs. In fact, our results are quite consistent with recent reports in which EDTA-exposed corneal endothelial cells were released from contact inhibition and subsequently proliferated [22]. Another study has shown that the cell-cell contact-induced inhibition is mediated, at least in part, by p27^kip1^ because the p27^kip1^ protein level is 20 times higher in confluent rat corneal endothelial cells than in subconfluent cells. In addition, the level of the p27^kip1^ protein is substantially lower in EDTA-treated confluent cells than in untreated control cells [23]. Although the link between PLZF and p27^kip1^ has not been determined, both genes presumably contribute to the contact inhibition of HCECs.

The cell adhesion molecules expressed in corneal endothelial cells are ZO-1, connexin-43, and cadherin [24-26]. Among these, the cadherins are major intercellular adhesion molecules that mediate calcium-dependent cell-cell adhesion through homophilic interactions [27]. It has been demonstrated that a breakdown in the cadherin-mediated cell adhesion activates a β-catenin-mediated intracellular signaling pathway, inducing the expression of a set of genes, including cyclin D1, c-myc, and c-jun, that are critical for cell proliferation and cell survival [28-30].

We have examined the expression of cadherin family mRNA in cultured HCECs and in vivo HCECs, and N-cadherin was found to be the major cadherin in HCECs. In addition, the expression of N-cadherin increased in proliferating HCECs before the expression of PLZF (Figure 5B). We have hypothesized that N-cadherin and its downstream signaling pathways are the candidate molecules involved in the regulation of the expression of the *PLZF* gene. Currently, experiments are being carried out in our laboratory to test this hypothesis.

The involvement of PLZF in suppressing the proliferation of cultured HCECs has been clearly demonstrated by infection of the *PLZF* gene in HCECs. The degree of the suppression was not as high as in murine 32Dcl3 cells, but considering an infection efficiency of 24% at an MOI of 100 with the Ad-GFP vector, an over-expression of PLZF led to a relatively high degree of suppression of the proliferation of HCECs. In addition, TSC-22 was found to be increased in HCECs which over-expressed the *PLZF* gene according to the DNA microarray analysis. TSC-22, a leucine zipper transcriptional factor, was found to be an immediate-early target gene of TGFβ_1_ [31] and has the characteristics of a suppressor of cell proliferation [32-34]. Moreover, TSC-22 binds to and modulates the transcriptional activity of Smad3 and Smad4, and it enhances TGFβ signaling by associating with Smad4 [35]. Evidence has been showing that TGFβ_2_ is present in high concentrations in normal aqueous humor [36,37], and it suppresses the proliferation of rabbit and rat corneal endothelial cells in vitro [7,38,39]. Thus, *TSC-22* might be involved as a target gene for PLZF in suppressing HCEC proliferation in accordance with TGFβ signaling pathway. This hypothesis, however, needs further investigation.

We have also examined the expression of PLZF mRNA in the corneal endothelial cells obtained from three patients with iridocorneal endothelial syndrome (ICE syndrome). Interestingly, the relative expression of the mRNA of PLZF in ICE syndrome was lower than in normal controls by real-time PCR. In particular, the expression of PLZF mRNA was undetectable in Chandler's syndrome (Figure 7). These findings suggest that the absence of the *PLZF* gene may lead to the abnormal proliferation of corneal endothelial cells, a hallmark of the ICE syndrome.

In conclusion, we have shown that the mRNA of PLZF was closely associated with cell-cell contact phenomenon of human corneal endothelial cells. Further studies are needed to clarify the correlation of the *PLZF* gene with the intracellular signals governed by TGF-beta and with the expression of the cell adhesion molecule such as cadherin. As PLZF is normally expressed in the human corneal endothelium, it can serve as a possible target to modulate cell proliferation. It may be possible in the future to treat patients with severe corneal endothelial damage if the key factor regulating the expression of the *PLZF* gene and the genes regulated by PLZF are identified.
